# Evolutionary Challenges to Humanity Caused by Uncontrolled Carbon Emissions: The Stockholm Paradigm

**DOI:** 10.3390/ijerph192416920

**Published:** 2022-12-16

**Authors:** Dmitry V. Boguslavsky, Natalia P. Sharova, Konstantin S. Sharov

**Affiliations:** Koltzov Institute of Developmental Biology, Russian Academy of Sciences, 26 Vavilov Street, 119334 Moscow, Russia

**Keywords:** emerging infectious disease, CO_2_ emission, greenhouse effect, global warming, climate change, IPCC, Paris Agreement, Kyoto Protocol, energy consumption, fossil fuel, evolution, ecology, host–parasite interaction

## Abstract

This review paper discusses the Stockholm Paradigm (SP) as a theoretical framework and practical computational instrument for studying and assessing the risk of emerging infectious diseases (EIDs) as a result of climate change. The SP resolves the long-standing parasite paradox and explains how carbon emissions in the atmosphere increase parasites’ generalization and intensify host switches from animals to humans. The SP argues that the growing rate of novel EID occurrence caused by mutated zoonotic pathogens is related to the following factors brought together as a unified issue of humanity: (a) carbon emissions and consequent climate change; (b) resettlement/migration of people with hyper-urbanization; (c) overpopulation; and (d) human-induced distortion of the biosphere. The SP demonstrates that, in an evolutionary way, humans now play a role migratory birds once played in spreading parasite pathogens between the three Earth megabiotopes (northern coniferous forest belt; tropical/equatorial rainforest areas; and hot/cold deserts), i.e., the role of “super-spreaders” of parasitic viruses, bacteria, fungi and protozoa. This makes humans extremely vulnerable to the EID threat. The SP sees the +1.0–+1.2 °C limit as the optimal target for the slow, yet feasible curbing of the EID hazard to public health (150–200 years). Reaching merely the +2.0 °C level will obviously be an EID catastrophe, as it may cause two or three pandemics each year. We think it useful and advisable to include the SP-based research in the scientific repository of the Intergovernmental Panel on Climate Change, since EID appearance and spread are indirect but extremely dangerous consequences of climate change.

## 1. Introduction

### Problem Overview

During the post-war era, the planet has been facing perpetual carbonization caused by several anthropogenic factors, mainly the omnipresent use of fossil fuels (coal, crude oil, distillates and natural gas) and organic chemicals [1]. Soils, waters, the atmosphere and the biosphere have all become significantly contaminated by the ever-growing levels of CO_2_—levels that were unfathomable even one hundred years ago [2,3]. Due to mass deforestation that has additionally increased carbon dioxide levels, many irretrievable changes in the biosphere have occurred that continue to happen each year at an elevated pace [4,5]. For humanity, as a part of the biosphere, this means serious environmental-related healthcare issues, from the emergence of new diseases to malfunctioning medical care systems because of the avalanche of these diseases [6,7].

The carbon emission crisis became obvious during the last 20–30 years, and today its dangerous consequences challenge public health in almost every region of the Earth [2,8]. These consequences involve both high-income countries and low- to middle-income states [8,9]. The challenges to public health directly or indirectly related to carbon emissions can be summarized as follows—they are all strongly interconnected:

1. *The direct influence of carbonization of the biosphere on human health*. The primary pollution by CO_2_ produced during fossil fuel combustion is accompanied by organic environmental pollution by petrochemicals. The latter mainly consists of contamination from leaded petrol and high-sulfur gas oil, from distilling crude oil in multilayer processing plants’ rectifiers and from driving vehicles without effectual engine gas utilization catalysts [10,11,12]. Carbon and carbon-related organic chemical pollution may cause a number of chronic diseases ranging from chronic obstructive pulmonary disease to immune disorders and even leukemia for populations residing near industrial areas [13].
2. *The imminent climatic catastrophe.* A gradual increase in the atmospheric temperature caused by the carbon dioxide oversupply (the so-called “greenhouse effect”) may lead to dangerous ecological consequences: melting of the Earth’s ice cover, heightening of the world ocean level, evolutionary shifts in biota and changes in climatic and weather patterns. Climate changes cause the redistribution of the water supply and removal in different regions of the planet. Some regions of the Earth have become exposed to droughts and wildfires, while others to floods [9]. In turn, this results in altered lifestyles of communities most affected by climate change [14,15,16,17]. Overcrowding of some areas, depopulation of others and the parallel augmented level of people migration, especially in low-income countries, worsen the level of hygiene, impair immunity and often cause epidemics. These epidemics have become customary for the definite regions of Africa, South/Southeast Asia and Latin America (cholera, malaria, plague, Zika disease, yellow fever, measles, smallpox, dengue fever, etc.).
3. *The world population’s uncontrolled growth rate.* Carbonization of the biosphere would not be a global issue of humanity were it not connected with overpopulation. As an ecological factor, global overpopulation of the Earth needs to be considered along with climate change. In 1971, Dennis Meadows and colleagues were the first to demonstrate the phenomenon of a double exponential growth of carbon emissions [18]. Humanity is multiplying at an exponential rate, and it does not limit its appetite for fossil energy consumption, which is increasing at an exponential rate too for a given cohort of individuals or companies [12]. As a consequence, we have a double exponential growth of carbonization of the Earth’s biosphere $C=fe^{ce^{at+b}+dt+j}+gt+h$, where *C* is overall carbonization, *t* time, and *a*, *b*, *c*, *d*, *f*, *g*, *h* and *j* coefficients [18]. Therefore, CO_2_ pollution itself is not the greatest hazard to the biosphere and society, but rather its indefinite and unmanaged double exponential growth.

4. *Global urbanization*. Overpopulation causes the global growth of urbanized environments, with megacities tending to prevail [19]. Megacities are city agglomerates spanning vast areas with urban infrastructure and building planning [20,21,22]. They have become the major loci of carbon dioxide emissions and biosphere perturbations. These agglomerates may span hundreds, even thousands, of miles and create unprecedented epidemiological risks for their inhabitants. The rate of infectious disease transmission may be exceptionally high in such densely populated regions due to the extremely high density of face-to-face contacts. The net incidence of diseases is also much higher than in low-populated areas because of the huge size of megalopolises (usually several million or even several dozen million people, e.g., Shanghai, Tokyo, Tianjin, Dhaka, Jakarta, Karachi, New York or Moscow).
5. *The phenomenon of emerging infectious diseases (EIDs).* These diseases represent a particular risk for global public health brought about by climate change and altered lifestyles of *homo sapiens* [23,24,25,26,27,28,29,30,31,32].

Certainly, limitless and unmanaged CO_2_ emissions are an evil for the biosphere, society and the very species *homo sapiens* as an evolutionary entity [33]. They are one of the main reasons for the appearance of novel, sometimes very hazardous, EIDs [34,35]. However, it is precisely these types of carbon emissions that we have been witnessing thus far [36,37,38,39,40].

Can we change them through decarbonization initiatives and thus lower the rate of EID appearance? This would definitely diminish the burden on public health in many countries.

Several theoretical frameworks have emerged since the early 1970s that connect public health with anthropogenic influences on the biosphere, including carbon emissions. Among the most popular, there are the aforementioned World 3 model proposed by Dennis Meadows [18,19], the concept “Spaceship Earth” advanced by Barbara Ward [41] and Richard Buckminster Fuller [42] (and many others) and the World in 2050 initiative being developed by Nebojša Nakićenović and his collaborators from IIASA [43]. The Stockholm paradigm (SP) is currently much less known to the scientific community.

In our narrative review, we aim at presenting and discussing the major provisions and heuristic value of the SP to evaluate the risk of EID appearance as a result of climate change and the changed evolutionary role of *homo sapiens* in the new ecological situation.

In our review, we will consider the SP from four perspectives:

- As a natural philosophical concept;
- As a methodological repository;
- As an instrument for practical research on EIDs in the situation of climate change;
- As an ethical framework.

The structure of our review reflects this multifaceted view. Section 2 gives a brief description of our reviewing technique. Section 3 discusses the philosophy of the Stockholm paradigm (the SP is a natural philosophical concept like Darwinism in its essence). Section 4 provides the theoretical core and base methodology of the paradigm. Section 5 considers the paradigm as a tool for practical implementation. Section 6 outlines the ethics of the paradigm. Section 7 infers the major consequences of the paradigm’s application to the evolution of humans. Finally, Section 8 summarizes the main conclusions of this review.

## 2. Materials and Methods

The SP is a relatively novel concept. It emerged from the consideration of the appearance of novel parasitic pathogens causing new EIDs every year. It mainly deals with evolutionary processes of inheritance system assemblages impacted by climate change [44,45,46]. The SP’s emergence was preceded by the highly increased rate of EID appearance due to climate change in the situation of the de facto ineffectiveness of the Kyoto Protocol and uncertain prospects of the Paris Agreement implementation in regard to restructuring public health systems. The paradigm was named after four generations of researchers at Stockholm University who contributed to its foundation. Daniel R. Brooks, now a Professor Emeritus at the University of Toronto, coined the name at a conference in Sweden in late 2013, and the name first appeared in the literature in the subsequent years.

This narrative review was composed according to the SANRA guidelines (Scale for the Quality Assessment of Narrative Reviews) [47].

To obtain the necessary bibliography, we screened the Science Direct and PubMed Central databases for original articles, reviews and books published from 1920 to 2022 in English. Several relevant works beyond this date range or outside of the English language requirement were collected arbitrarily, e.g., several classical works on evolution were included by choice.

The automatic search was performed in accordance with the following keywords (collocations): Stockholm paradigm (Group 0); CO_2_ emission, carbon dioxide emission, carbon emission, carbonization, greenhouse effect, global warming, climate change, fossil fuel, ecology, environment, overpopulation, Spaceship Earth (Group 1); evolution, host, parasite, parasite paradox, emerging infectious disease, inheritance system, species, fitness space, (co-)speciation, (co-)phylogeny, generalization, specialization (Group 2). The items that were included in our review were identified using the following criteria:

Option 1

(A) At least four key words in Group 1 were found (in the keyword list, title, running title, abstract or the text in cases where the full text was available);
(B) At least three key words in Group 2 were found.

Option 2

(A) Group 0 was presented;
(B) At least two key words in Group 1 were found;
(C) At least two key words in Group 2 were found.

From the set of 1869 works, we manually selected 102 items (5.46%) that offered a direct or implicit discussion of the Darwinian perspective of evolution. The inclusion procedure was carried out manually by two reviewers (D.V.B. and N.P.S.), independently of each other. The third reviewer (K.S.S.) helped to resolve issues of inclusion through discussions.

After the arbitrary addition of several classical evolutionary literature sources, we retrieved 123 items.

The theoretical core of the SP is largely developed by D. R. Brooks and co-authors. The works of the research teams associated with Professor Brooks are the first part of the bibliography.

Since the SP is effectively an evolutionary paradigm, we used evolutionary, analytical and comparative approaches. We compared the SP’s theoretical foundations with the works of the most prominent evolutionists (Darwinists, neo-Darwinists, orthogeneticists and neo-Lamarckists). These works comprise the second part of the bibliography.

The third part is formed by works where the SP is discussed as a practical tool for appraising the risk and predicting the characteristics of new pathogens, as well as coping with EIDs brought about by climate change.

The fourth part consists of publications of different research teams and individual authors unrelated to the SP who study the environmental and public health impacts of climate change.

## 3. Philosophy: Evolutionary Essence of the Stockholm Paradigm

### 3.1. Difference between the Stockholm Paradigm and Other Environmental Concepts

The authors of the SP consider the phenomenon of EIDs in an evolutionary—to be more exact, Darwinian—way. This attitude implies considering the whole bundle of questions listed in the Introduction (1) together and (2) as evolutionary factors that may redefine the life, activity, development and fate of *homo sapiens* as a species.

The SP does not focus merely on EIDs, the biosphere or climate change. It utilizes a systemic approach and argues that the increasing rate of EID occurrence is related to the following factors brought together as a unified issue of humanity: (a) carbon emissions and consequent climate change; (b) resettlement/migration of people with hyper-urbanization; (c) overpopulation; and (d) human-induced distortion of the biosphere. This sets the SP apart from most of the modern ecological theories and practical/political initiatives that try to address one separate issue at a time.

Specifically, from the SP’s viewpoint, it is vain to restructure a public health system in a given country or even global health protocols by only trying to solve any of these problems independently of each other. The problems need to be solved, at least partially, simultaneously—this is the main admission of the SP. Therefore, the SP argues that a mere agreement on a decarbonization protocol, e.g., a protocol adopted by the United Nations Framework Convention on Climate Change (UNFCCC), however effective it might be for the sole purpose of reducing carbon dioxide emissions, will not automatically and completely decrease the hazard of EIDs to public health. However, the SP analyzes, in detail, environmental changes of an anthropogenic origin that have led to the instability of many public health systems and their vulnerability to the ever-growing number of EIDs. Consequently, the SP can be a valuable tool for establishing the connections between future carbon emission scenarios and healthcare system adjustments.

### 3.2. Adherence of the Stockholm Paradigm to the “Survival of the Adequate” Evolutionary Principle

The SP’s creators built their conceptual framework around classical Darwinism. The modern theory of evolution mainly consists of Darwinian, neo-Darwinian, neo-Lamarckian and orthogenetic approaches. The main difference between Darwinism and non-Darwinian approaches (neo-Darwinism, neo-Lamarckism and orthogenetic concepts) is that the latter take the “survival of the fittest” principle as a basic provision, while Darwinism does not do this.

Anthropogenic perturbation of the biosphere—remembering that carbon emissions are its main factor—entails the following evolutionary hypothesis shared by almost all non-Darwinian evolutionists. *Homo sapiens* occupies the highest place in the evolutionary ladder, as it is at the very top of all food chains. Therefore, humans as a species are the fittest of all living systems that have ever inhabited the planet. Consequently, humans may not strive for a battle for the biosphere, the victory of which could give humans new means of their own survival, e.g., novel techniques of preventing, controlling, treating or coping with EIDs. In short, humans will survive under any environmental conditions. Such a view necessarily implies that the biosphere will transform into the “neglected other” for society [39,47].

The SP as a Darwinian framework adheres to a different principle, namely, “survival of the adequate”, introduced by Charles Darwin himself in his major work *The Origin of Species* [48]. The SP’s authors warn that there is a non-zero chance of *homo sapiens* not surviving a future catastrophe of EIDs brought on by climate change. Therefore, humans must begin to manage future scenarios of carbon emissions right now.

After Herbert Spencer took over with his *Principles of Biology* [49,50,51], where he substituted the “survival of the fittest” principle for Darwin’s initial “survival of the adequate,” it was a common view that the classical Darwinian evolutionary model poorly works—if works at all—in evolutionary ecology. Vernon Kellogg proclaimed Darwin dead (“Darwin is dead, let’s get modern”), stating that Darwinism was of critical historical importance but little scientific value [52]. The SP demonstrates that these assumptions may be rash and, speaking plainly, incorrect, while classical Darwinism can still be a valuable evolutionary concept to be included in future decarbonization philosophy.

However, readers may question to what extent the Darwinian foundation of the SP is justified. Specifically, this question will be, *how can we combine Darwinism and fundamentally changed ecological environments where global humanity has already altered the whole ecosystem of planet Earth?* Indeed, regarding the climate shift, the enormously increased rate of population growth—with 13–15 billion people expected by 2100 [53]—huge migration and tourist currents, ever-augmenting contiguity of humanity with the remainder of “wild” nature perturbed by humans and rising super-megacities with unprecedented epidemiological situations, all of these phenomena determine our New Brave World, which was obviously unknown to Darwin.

The SP provides an answer. Recalling John Maynard Smith’s evolutionary metaphor, the “Gambler’s Ruin” [54,55], the SP’s authors invite readers to look into how Darwinism and modern environmental perturbations can be conciliated [44]. Introducing game theory to evolutionary biology, Maynard Smith was once occupied by demonstrating that evolution cannot foresee future perturbations, and therefore, it does not necessarily produce the best of all possible outcomes, in contrast to what non-Darwinians claim and believe in. A gambler who always raises his bet and does not reduce it when he loses will go bankrupt even if he has a positive mathematical expectation on each bet.

In evolutionary terms, this means that evolutionary processes are not perfect. An inheritance system may cope with the uncertainty of the current ecological environment today, but it may become extinct tomorrow when the conditions change abruptly or in ways that will not allow the inheritance system to adjust or adapt. Inheritance may be regarded as “anticipatory” in the sense that living systems “anticipate” that there will be no drastic change in conditions in the nearest future. For most evolutionary cases, this works and ensures the survival of organisms. However, we cannot but reiterate that this does not guarantee the eternal survival of a given species, in our case, *homo sapiens*.

If evolution is imperfect and sometimes cannot even prevent disastrous outcomes for some species, contrary to what non-Darwinians say, how can humanity rely on its highest place in the evolutionary ladder? Maynard Smith’s metaphor, the “Gambler’s Ruin”, perfectly describes the situation in which humanity is plunging with uncontrolled carbon emissions that are rushing to the +2.0 °C global warming level. Humanity increases its bet on fossil energy consumption, yet it does not manage the risk of climate change and EIDs, i.e., the risk of its own extinction as a species.

### 3.3. Arguments of the Stockholm Paradigm against Non-Darwinian Evolution Theories

What considerations does the SP have to oppose the non-Darwinian understanding of our evolutionary future?

The neo-Darwinian framework, largely based upon the ideas of Herbert Spencer [48,49,50,51], Vernon L. Kellogg [52], Theodosius Dobzhansky [56,57,58,59,60], Julian Huxley [61,62,63], Stephen Jay Gould [64,65], Robert H. MacArthur [66,67] and Charles E. King [68], has a weaker potential than the SP to explain why EID appearance and incidence have risen sharply in the last 20–30 years. Let us consider neo-Darwinism, orthogenetic theory and neo-Lamarckism in detail.

Neo-Darwinism underestimates the evolutionary and existential risk for humanity caused by uncontrolled and ever-growing carbon emissions, for the following reasons:

(a) It includes pan-adaptationism as its core foundation (the strong but mainly ungrounded belief that the perfect adaptation of species is a cornerstone of evolution);
(b) It understands evolution in terms of an inflexible space–time duality;
(c) It admits the presence of co-evolution (a principle where co-phylogeny always involves co-speciation) and uses the notion of co-evolution to explain almost every interspecies relationship;
(d) It admits that the nature of conditions is much more important than the nature of organisms;
(e) It comprehends natural selection as a universal creative instrument and the uttermost reason for every evolutionary process in the biosphere;
(f) It deduces functions of an organism from evolutionary conditions unequivocally;
(g) It replaces the principle of conflict resolution with never-ending conflict as a raison d’être for evolution.

In addition to these limitations of neo-Darwinian theory that prevent non-Darwinians from having an adequate estimation of the evolutionary risks of EID appearance due to climate change, orthogeneticists believe in the “rightest” and “most progressive” evolutionary diversification, the “adaptive radiation from primitive and central types,” a view widely held after Henry Osborn [69]. In the context of EIDs, the orthogenetic view would allow the appearance of a better *homo sapiens* in the future that will be inherently immune to most EIDs, as it itself creates the environmental conditions to live in. In short, orthogeneticists believe that no species, including *homo sapiens*, can destroy itself by creating environmental conditions that, in time, would exterminate the very species.

In a paradigmatic context, the authors of the SP problematize neo-Darwinism, especially the neo-Darwinian approach to co-evolution. They call to return to Darwin, whose views were neglected and forsworn by neo-Darwinians:

There is a dark side of this success story. The notion of conflict resolution has been replaced by never-ending conflict as the raison d’etre for evolution and for evolutionary biology. In keeping with the focus on never-ending conflict, the scientific literature began to fill with aggressive anthropomorphisms–selfishness, cheating, attacking, defending, invading, sneaking, arms races, aliens, defense–counter defense, enemies, deceit, sexual conflict. In parallel, there were many personal attacks on almost any new idea that deviated from the simple core… What neo-Darwinism replaced was Darwinism.

[44]

The SP’s authors argue that the neo-Darwinian understanding of evolution as a superposition of ecology and genetics without resolution of conflicts may be a perspective approach only when we know the border conditions and laws of dynamics of the development of all inheritance systems in a given geographical region. In the case of the strong and ever-increasing anthropogenic impact on ecosystems, with changing laws of inheritance system development dynamics, the neo-Darwinian approach is in danger.

Neo-Lamarckists admit that the ability to cope with any evolutionary threat, including infection, will be passed on to the progeny by the parent, a concept that reached our days from the 1920s–1930s when it was first formulated by William McDougall [70,71] and Arthur Ward Lindsey [72]. Modern neo-Lamarckians would argue that altered environmental conditions, which place humans in a state of survival, will determine the inherited ability of future generations to cope with EIDs.

As we see, neither of these assumptions (based on neo-Darwinian, orthogenetic or neo-Lamarckian views) can be witnessed now. Indeed, the crisis of carbon emissions is obvious (it will soon hit the two-degree level), and the proliferation of EIDs is on the rise (with COVID-19 as possibly the best recent example), while humans continue to be extremely vulnerable to the novel EIDs brought about by anthropogenic influences on the biosphere, but to which human immunity is not yet accustomed.

This questions the practical usefulness of neo-Darwinian, orthogenetic and Lamarckian concepts for the purpose of integration into decarbonization strategies, as well as their heuristic and predictive force.

Instead, the attitude of the SP is to recognize that in the twenty-first century, humanity as an evolutionary entity (an inheritance system) has entered an unstable state: the more it proliferates and consumes fossil energy with carbon dioxide emissions released into the biosphere as an inevitable side effect, the closer it is to its own extinction [26,28,73].

### 3.4. Genetic Relation to Other Modern Ecological Concepts

The SP is genetically related to several modern ecological concepts. The stratagem of the SP of reassessing and reasserting the value of Darwinism in using evolution as a means of humanity’s survival in completely new ecological environments demonstrates a resemblance to the evolutionary concepts of John Maynard Smith [74,75] and Koichiro Matsuno [76,77], as well as the concept of life of Tibor Gánti [78,79]. Salvatore Agosta and Daniel Brooks are certain of the following in their book, *The Major Metaphors of Evolution: Darwinism Then and Now*:

Putting evolution to work for humanity is about recognizing that the fundamental resource in the biosphere for coping with change is the potential stored in the evolutionary commons. For our own well-being, our economic policies and strategies should reflect this… *Darwinism Then and Now* is fundamentally a story about coping with change by changing… The evolutionary context of global climate change involves two variables: *Ecosystems*, upon which we depend for survival; and *Us*, who have the capacity to alter those ecosystems to such an extent that the continuation of humanity will be jeopardized.

[44]

An unobvious link connects the methodology of the SP with that of *The Limits to Growth* [18,19] by Dennis Meadows and the evolutionary concept of Wolfgang Sassin [9,10,11,12,15,16,17].

Dennis Meadows, the founder of The Balaton Group created in 1982, and his team of researchers initially argued that strict environmental preservation and ecological control aimed at achieving the sustainable development of humanity might help nature to return to evolutionary processes that had been taking place before humanity became a global phenomenon [18]. 

In the 2010s, Meadows evidently switched from the dogma of sustainable development to recognizing a crucial role of humans in any further evolutionary processes on Earth. He introduced the term “human-defined evolution” and renounced his unconditional belief in sustainability as a means of humans controlling and “protecting” evolution [19]. According to Meadows, “human-defined evolution” is the intervention of humans in biota to adjust the species to their own needs and make some of their new traits inheritable. This evolution, as Meadows argued, would inevitably move away from nature towards “culture”, allowing human-modified species to survive, and cutting away the rest of the “unnecessary” species. Dennis Meadows believed that Darwin’s “survival of the adequate” would, to a certain degree, transform into the “survival of those necessary to global humanity”. Such an intervention in evolutionary processes may endanger not only multiple species but also humans as a species [19]. It is not difficult to see the multitude of EIDs that appear as a side effect of “human-defined evolution.”

Thirty years after the publication of *The Limits to Growth*, Wolfgang Sassin admitted that humanity could not re-create evolutionary environments of the past [9,11]. When Darwin was writing his *Origin of Species*, the world population was around one billion people and, according to Sassin, nature was “self-controlling” [9,11,12]. Those conditions do not exist any longer [10]. Humans have converted nature into “culture” completely, as finite natural resources are being exhausted and natural “recycling” processes modified [12]. The very essence of the global consumerist lifestyle of *homo sapiens* makes the protection of pristine nature, where “classical” laws of evolution would explain natural selection, biodiversity, speciation, adaptation, inheritance and variation, impossible, as no return to pristine nature in the modern Anthropocene is feasible [12]. Wolfgang Sassin expounded the following:

In the contemporary political discourse, we may observe… a naïve and self-contradictory green argument: it is not the global spread of the modern lifestyle but only material growth based on fossil fuels that endangers nature, from which *homo sapiens*, as a biological product of evolution, cannot detach itself. Nature must, therefore, be strictly protected for the sake of human beings, who continue to multiply and at the same time claim well-being as an unconditional human right that must be extended in every respect.

How can one realize the consequences of such utopian a vision, the questioning of which is not only considered immoral, but the analysis of which is to be made legally difficult, if not prevented, by sanctions?

[12]

Obviously, rolling back to the revival of former conditions of natural selection and speciation in the biosphere by cutting the consumption of global humanity is not an option. What we need now is a new theoretical understanding of how *homo sapiens* may survive as an evolutionary entity in the situation of EID appearance caused by climate change. The SP implicitly aims at proposing such an understanding.

## 4. Methodology: The Stockholm Paradigm’s Explanation of the EID Phenomenon

### 4.1. Theoretical Core of the Stockholm Paradigm

The authors give a broad definition of the SP:

The integration of capacity and opportunity, and the complementary oscillations in fitness space arising from perturbations in the conditions is the Stockholm Paradigm.

[44]

To make the concept more understandable, we may say that the SP is an attempt to integrate spatial (taxon pulses) and functional oscillations between being explorers and exploiters of the surroundings in the fitness space. More specifically, the paradigm is a theoretical framework that explains relationships between (1) expansion and isolation; (2) exploration and exploitation; and (3) generalization and specialization of species. External perturbations cause cyclic switches between expansion/exploration/generalization and isolation/exploitation/specialization. The SP tries to account for these switches and predict shifts in biogeographical boundaries.

The paradigm is constructed in a flexible way, as it admits both generalization and specialization of species, the two processes that may take place separately and independently. Thus, the paradigm helps to escape a blind alley of the pan-specialization of organisms—it operates the notion of evolutionary potentials. Basically speaking, the SP re-considers our understanding of co-speciation by problematizing the universalism of neo-Darwinian co-evolution theory:

From the perspectives of the Stockholm Paradigm, therefore, the very phenomenon of coevolution may be illusory.

[44]

In the classical evolutionist approaches of the twentieth century (neo-Darwinism, neo-Lamarckism and orthogeneticist views), there is a concept of the co-evolution of inheritance systems. It admits that the closer the relations between two species, the higher the level of co-evolution between them. Therefore, it suggests that co-phylogeny inevitably leads to co-speciation. In the SP, it is supposed that co-speciation may be a rather uncommon result of co-phylogenetic development.

Different organisms have different abilities to fit in the same ecological environment. After external perturbations happen in an ecosystem, the fitness space within the given biogeographical boundaries becomes sloppy. The stronger the perturbation, the sloppier the fitness space after it.

Regarding these perturbations, different inheritance systems will adapt to new conditions within these biogeographical boundaries in different ways. Some will do it effectively (the chance of survival is high), while others will not (conversely, the odds of extinction are high). For example, some parasites may expand their host range, and some non-parasitic species (carnivorous or herbivorous) may add new feeding opportunities to their common diet. The others will do it to a small degree or will not do it at all. Therefore, after perturbations have subsided, new biota and new ecosystems will arise in a given geographical area. A definite level of co-phylogeny may be found between the old and new sets of inheritance systems living in old and new ecological environments. The SP emphasizes that phylogeny, even the phylogeny of species closely related in the old ecosystem, will be very rarely tracked completely in the new ecosystem, i.e., co-speciation is an occasional outcome and not the rule, in contrast with what the classical evolutionary concepts state. Therefore, the SP defies the classical evolutionary paradigm of co-speciation we are accustomed to, which states a maximal level of co-evolution in newly formed ecosystems.

The SP contains the following core theoretical concepts:

- Understanding of the paramount significance of the nature of an organism;
- Ecological fitting in an inherently sloppy fitness space;
- A slightly modified oscillation hypothesis (with several provisions of the taxon pulse hypothesis);
- Modified geographic mosaic theory of co-evolution;
- Conflict resolution for individual species and diversification of interspecific associations.

Let us consider the constituents of the SP in detail.

1. *Geographic mosaic theory of co-evolution*. The geographic mosaic theory of co-evolution, ascending to the works of John Norton Thompson [80,81,82,83,84,85], can account for possible interactions between species specialized in the fitness space and circumscribe the boundaries of these processes. We may recall that the neo-Darwinian concept of co-evolution suggests that co-phylogeny almost always means co-speciation. In fact, this is not strictly and not always true. In reality, co-speciation is only one possible co-phylogenetic outcome [27]. Thompson’s geographic mosaic theory of co-evolution convincingly explains how generalists become specialists, but it does not explain how new generalists evolve from old specialists. The theory has a flaw in its postulation of the following two points: (1) the strict division between generalists and specialists, and (2) the adherence to the “almighty faculty” of co-evolution.

Indeed, co-evolution leads to the development of highly specialized organisms. From Thompson’s viewpoint, in time, organisms will specialize more and more. Consequently, their evolutionary potential shrinks as the host range (for parasites), habitat or diet (for non-parasitic species) contracts. Co-speciation for parasites with the existing hosts can become very strong, and their ability to switch to new hosts, explore new habitats and resources or adjust to new diets diminishes.

However, without the possibility for specialists to become generalists, there can be no way for survival in the case of serious external perturbations when the fitness space becomes extremely sloppy. The SP adds flexibility to the geographic mosaic theory of co-evolution, switching from static to dynamic concepts. The paradigm does not operate the static concepts of specialists and generalists. Instead, it admits that there are species that can be dynamically specialized or generalized in the sloppy fitness space. Thompson’s theory leads to the possibility of conflict resolution, a key element in the SP.

2. *Oscillation hypothesis.* For species, it is customary to exist in associations that exploit the fitness space. They are maximally specialized in those conditions. Regarding the external perturbations of ecosystems, the fitness space becomes increasingly sloppy and the ecological relationships in it change. Species switch from being specialized to generalized (exploration). The greater their capacity for ecological fitting, the more they utilize new possibilities in the sloppy fitness space. To a certain degree, when ecological conditions allow an ecosystem to return to a state closer to equilibrium, generalization transforms to new specialization (return to exploitation).

Oscillations between exploration and exploitation occur any time when radically changed environmental conditions shift an ecosystem from its balance. Changes between increasing and decreasing the range of ecological interactions in relation to the sloppiness of the fitness space form the core of the oscillation hypothesis, which was first formulated by Niklas Janz and Sören Nylin [86,87].

It is noteworthy that oscillations do not happen evenly throughout time and evolutionary history. For parasites and their hosts, the oscillations are “squeezed” in periods of increasing and decreasing the host range—it is for this reason that Janz and Nylin introduced the term “oscillations.” An host range increase provides allowance for future possible imperfections, discrepancies and disparities in host–parasite co-phylogeny during the periods of host range contraction. Moreover, it should be stressed that the fitness space is inherently sloppy, with the idea first being advanced by Salvatore Agosta [14,45,46].

3. *Taxon pulse hypothesis.* The taxon pulse hypothesis initially proposed by Terry Erwin (1979) explains augmented connectivity in the geographic fitness space when external perturbations occur in ecosystems. The taxon pulse hypothesis is connected with the theory of taxon cycles introduced by Edward Osborne Wilson [88,89,90,91] and further developed by Joan Roughgarden [92].

In contrast with taxon cycles, taxon pulses do not require effusion from one center or place of origin, thus, allowing the various sources of origin of biota in a given territory that might contribute to its diversity at different times. Taxon pulses take place in expansion and isolation phases. Breaking down existing evolutionary barriers and creating new ones are determined by the nature and length of external effects on ecosystems and biotas. Within the SP, the taxon pulse hypothesis along with the oscillation hypothesis accounts for the variety of inheritance systems in a given geographical space.

Indeed, taxon pulses brought about by external influences on ecosystems are the factors determining the geographical diversity of biotas made up of species closely associated with each other. Taxon pulses are historically repetitive. They produce different biotas from species that might co-exist and be associated with each other during different periods of time. Therefore, taxon pulses and oscillations are complementary evolutionary frame systems, and the oscillation hypothesis and taxon pulse hypothesis are complementary concepts within the SP. However, the SP eschews the physical space–time dichotomy. Daniel Brooks explained the evolutionary duality implied in the new SP in the following words:

Evolvable life embodies an irreducible Space-Time. Perspectives that space and time are separate independent variables [a dichotomy] with no connection to the inherent dynamics of living systems are neo-Darwinian and not Darwinian.

The relevant duality (not dichotomy) is the Nature of the Organism (inheritance space-time) interacting with the Nature of the Conditions (geographic space-time setting the context for inheritance space-time [aka fitness space]). This is the complex systems (Darwinian) perspective of interactions between the properties of the system and the properties of the surroundings rather than the traditional physics-based view that the state of the system at any one time is the result of the influences of various external variables alone (i.e., the neo-Darwinian view).

(Brooks, unpublished)

Different species have different fitting capabilities. Therefore, each species utilizes new advantages in the sloppy fitness space in the situation of external perturbations, to different extents. Moreover, the very means of utilization may differ. This provides a huge number of combination outcomes in biotas and ecosystems. There may be some degree of co-phylogeny between new and old evolutionary communities, but this co-phylogeny does not generally lead to co-speciation.

### 4.2. Resolution of the Parasite Paradox

How does a new EID appear as a result of an anthropogenic-carbonizing biosphere, from the SP’s point of view?

Climate change leads to the appearance of geographic zones with altered environmental conditions. These zones are spread unevenly across the globe, and climate change affects different parts of the biosphere (viz., biocoenoses and species assemblages) unequally. Some species in these zones are more vulnerable to human-induced perturbations, while others are much less vulnerable. The most vulnerable species either migrate to different territories less affected by climate change and the relocation/migration of people, or die. More stable species begin to find new ways to survive. Evolutionarily speaking, their fitness space becomes sloppy [93]. This means that these species—not organisms—cannot survive with their former customary food. They commence to add different opportunities to their common diet.

Around sixty per cent of all species on Earth are parasites, i.e., they live, proliferate or feed on or at the expense of different organisms (hosts) [94,95,96,97,98,99]. Among the parasitic world, especially dangerous to humans, there are viruses, bacteria, fungi and protozoa. Many of these species parasitize animals. By now, humanity has been struck by around 1400 parasitic species, but—strangely enough—less than 10 per cent of known parasites are well documented, described and studied [100].

With climate change, overpopulation and the mass resettlement/migration of people, a number of animal species migrate to the areas less affected by the anthropogenic influence on the biosphere. Parasites that live and feed on these animal hosts start to search for other hosts that may give them new resources to survive in the sloppy fitness space in the old territory, if the climatic, temperature, humidity and UV conditions are still appropriate for them there. As a major rule of thumb, this new host is *homo sapiens*, as it is often the most numerous species in the areas perturbed by it (e.g., in megacities and peri-urban areas that encompass yesterday’s regions of wild nature). The SP explains the appearance of novel EIDs as a result of the host switching of former zoonotic parasites from animals to humans [101,102]. Some of the parasites expand their host range, which may include both animals and humans [103,104,105,106].

The host switching that may take place as rapidly as in a few weeks or even days is incomprehensible and unexplainable for the non-Darwinian evolutionist approaches we have discussed above. They firmly adhere to the principle of co-speciation. According to it, if a parasite has been causing a zoonotic disease for at least several centuries, it has reached a perfect state of co-evolution with the animal(s) on which it has parasitized. This view can be found in the works of N. Janz [86,87], MacArthur and Wilson [90]. From their viewpoint, in time, animal parasites specialize more and more. Consequently, their evolutionary potential shrinks as the host range and diet possibilities contract. Co-speciation of animal parasites with the existing animal hosts becomes very strong, and their ability to switch to a new host (e.g., *homo sapiens*), explore new habitats and resources or adjust to a new diet diminishes [85].

The articles of Puschendorf et al. [103] and Hallas et al. [104], which use the SP as a practical instrument of prediction, explain these limitations of the non-Darwinian perspective on host–parasite relationships in detail.

According to the SP, a definite level of co-phylogeny may be found between the old and new sets of inheritance systems living in old and new ecological environments. The SP emphasizes that phylogeny, even the phylogeny of mammal hosts and parasites closely related in an old ecosystem, will be very rarely tracked completely in a new ecosystem, i.e., co-speciation is an occasional outcome and not the rule, in contrast with what non-Darwinians assert. As we noted above, the SP defies the non-Darwinian understanding of co-speciation that requires the maximal level of co-evolution in newly formed ecosystems.

The statistics of novel EID appearance prove that the non-Darwinian assumption works poorly. Humanity constantly continues to experience new diseases whose causative agents have never had any co-evolution with *homo sapiens*.

Here, the SP resolves the long-standing *parasite paradox* that non-Darwinian theories could not tackle.

If we consider not one parasite but a group of parasites, we can witness an emergence of related epidemics almost at the same time, as was the case with H5N1 (“bird influenza”) and H1N1 (“swine influenza”) [105,106,107,108,109]. With climate change, *homo sapiens* enters a new stage of its evolutionary fate. Without any co-speciation or evolution of the host utilization capabilities of EIDs’ causative agents, *homo sapiens* becomes a target for an unprecedented number of parasites. During host switches, parasites retain their phylogenetic properties determined by phenotypic (contextual) and situational flexibility without any following human phylogeny [39].

In the situation of the pernicious influence of carbon dioxide emissions on the biosphere, a growing number of zoonotic pathogens change their hosts and shift to humans [23,24,25,26,27,28,29,30]. The H5N1, H1N1, SARS-CoV 2002–2004, SARS-CoV-2, Ebola and Nipah viruses are a few most dangerous parasites that stroke humanity during the last two decades.

The SP argues that a zoonotic pathogen can swiftly switch to a human in a densely populated urban or peri-urban region with biota damaged by carbon emissions and other anthropogenic factors. This may cause an outbreak of a novel EID that is still unknown to humanity. Further, a local outbreak of an EID can easily transform into a pandemic, as probably happened with COVID-19 [31,32].

## 5. Instrument for Practical Research: Epidemiology, Genetics, Ecology, Evolution

### 5.1. COVID-19 Case

SARS-CoV-2 is a sad recent example of a zoonosis transforming into a human disease. The SP explains why it happened.

According to the most common version of its origin, the predecessor of the SARS-CoV-2 virus was initially spread in wild nature reservoirs such as bats (natural host) and pangolins (intermediate host) [29,30,32,110]. As a result of humans’ close contact with these reservoirs and destruction of environmental barriers, probably by eating the raw meat of these animals (a traditionalist Chinese delicacy), the predecessor could mutate to SARS-CoV-2 and jump to humans as its new host [101]. A total of 33 out of 585 environmental samples from the Huanan Seafood Market (Wuhan) showed evidence of SARS-CoV-2 [25]. These samples were collected, for the most part, from the market’s western ward, where alive or slaughtered specimens of wildlife had been sold. Recently, Jesse D. Bloom advanced an unusual hypothesis based on studying SARS-CoV-2 sequences from early in the Wuhan epidemic that has been deleted from the NIH’s Sequence Read Archive, which states that SARS-CoV-2 presence may have been reported for the first time not from the specimens of the Huanan Seafood Market, but from wild bats in southern Chinese provinces not associated with the Huanan market [111].

However, both seafood-market-related and -unrelated theories of the novel coronavirus origin agree with each other that it was a host switch of a parasite that led to the pandemic.

SARS-CoV-2 might be a mutated version of the virus common for *Rhinolophus affinis* bat species. This assumption is based on the 96.2% genomic homology between SARS-CoV-2 and BatCoV RaTG13. BatCoV RaTG13 diverges at the receptor binding domain of the S-protein, which suggests that it may not bind efficiently to the human ACE2 receptor and could have evolved in a different animal species before infecting humans [31,111]. Previous studies of other human coronaviruses that share a considerable part of their genome with SARS-CoV-2, viz., SARS-CoV and MERS-CoV, concluded that Himalayan palm civets and dromedary camels might act as intermediate hosts [99,110]. In South Asia, Southeast Asia and the Far East, humans are in constant close contact with these species, which may have added to the local outbreaks of coronavirus-caused EIDs during the last 20–30 years.

Whether or not climate change was the initial factor that triggered the chain of evolutionary events that finally led to the COVID-19 pandemic is a disputable matter. However, even now, we can recognize that the host switching of SARS-CoV-2 from bats and pangolins (if the disease did appear due to the host switching of SARS-CoV-2) to humans was not an occasional or unpredictable evolutionary event.

The SP predicted that the probability of a pandemic such as COVID-19 was substantial merely a year before the 2020 events, on the basis of analyzing the occurrence rate dynamic of local EIDs, which was constantly growing in the Far East, South Asia and Southeast Asia from 2002 to 2004 onwards, i.e., since the time of the appearance of SARS-CoV [39]. In contrast with COVID-19, SARS-CoV-related atypical pneumonia disease (2002–2004) did not transform into a pandemic. This was not due to a lower reproduction number (infectivity) or the commendable ability of local authorities to contain SARS-CoV at its very nidus, but rather because the biosphere (biocoenosis) of the Far East, especially southern China, where both viruses were allegedly detected for the first time, was drastically changed during the 16–18 years that passed since the emergence of SARS-CoV as a causative agent of human disease.

Due to (a) the mega-industrialization of the region based on fossil energy, (b) the overpopulation of the region, (c) the resettlement of people caused by climate change and floods, (d) the increased urbanization of the region, (e) the enhanced tourist activity of people and (f) the altered migration routes/behavior patterns of animals, the population of the region and newcomers (tourists and working migrants) came into much closer contact with local wild nature than in 2002–2004. By 2020, there was already almost no truly “wild” nature in southern and eastern China, including Hong Kong and the Pearl River Estuary. Additionally, this “artificial” nature was perturbed by human activity to the extent where Pandora’s box was opened [112,113,114]. Containing the outbreak of a novel EID was not carried out efficiently, as the public health systems of all countries were unprepared for a scenario like this.

### 5.2. The Risk of Other EIDs

During the last two decades, humanity has faced more than a hundred EIDs. Some of them have relatively high (0.5–1.5%) and high (more than 1.5%) mortality [115,116,117,118]. Among these EIDs, there are notorious SARS-CoV, Ebola, H1N1, H5N1, Nipah and, finally, SARS-CoV-2 viruses. All of them were once zoonotic pathogens and infected animals. The SP helps to estimate the possible aggressiveness of a novel pathogen (now human, but formerly zoonotic) and, therefore, ponder on the mortality it may cause in dense (urban areas) and sparse (villages) populations. In this chapter, we shall provide a few examples.

Since the SP is an emerging concept, not much practical research work has been conducted based on it thus far. However, there are important publications that prove its practical usefulness as a tool for assessing the risk of EIDs connected with climate change. Among them, there are the following notable contributions of scientific groups working at the University of Toronto (Toronto, Canada), the Federal University of Parana (Curitiba, Brazil), the Institute for Evolution (Budapest, Hungary), the University of Wisconsin-Madison (Madison, USA), the University of Illinois at Urbana-Champaigne (Champaigne, USA) and Virginia Commonwealth University (Richmond, VA, USA):

1. Walter A. Boeger et al. created a mathematical modeling platform for the SP to reveal the origin of the Omicron strain of SARS-CoV-2 [119]. On the basis of their modeling, the authors deduced the retro-colonization of humans by this strain from an unidentified mammal that was initially infected by humans. This result was subsequently grounded by additional empirical data obtained by other researchers [120,121,122]. Within their model, Boeger et al. proved the presence of host oscillations for SARS-CoV-2, one of the core elements of the SP’s theoretical framework.
2. In their model, Eric P. Hoberg et al. sustained that SARS-CoV-2 would not have switched from bats to other mammals in the absence of biocoenoses that are overcrowded, carbonized and distorted by humans, which acted as both mediators and super-spreaders of the novel infection [123]. Most importantly, the authors demonstrated that humans destroyed the natural borders of the biocoenoses, which delineated the natural reservoirs of SARS-CoV-2.
3. In their computational model, Elvira D’Bastiani et al. investigated how the intensity of host switching, assumed to depend on the phylogenetic distance between hosts, affects the ecological and evolutionary patterns of parasites under different climatic conditions: “normal”, and changed under anthropogenic influence [124]. This model allows one to link the intensity of exploration and colonization of new hosts by parasites with mutations and genetic drift. A model like this can be a valuable tool for appraising possible scenarios of EID appearance and further development caused by climate change.
4. Elvira D’Bastiani et al. performed another numeric calculation to establish connections between taxonomy, host body size and ecological opportunity in host–parasite networks [125]. The authors showed that the use of the metrics of connectance, nestedness and modularity to characterize the network structure permits one to assess the anthropogenic perturbation of an ecosystem semi-quantitatively or even quantitatively. Their model allows one to predict zoonosis–human disease switches with a commendable degree of exactness. Their predictions were supported by empirical data collected in mammals.
5. Sofia G. Feronato et al. advanced a PC modeling algorithm that connected three populational parameters of parasitic pathogens (reproduction rate, rate of novelty emergence and propagule size) with the intensity of the colonization of new hosts by these pathogens in the situation of the anthropogenic perturbation of biocoenoses [126]. The results of the algorithm proved that, contrary to the common view, the rate of novelty emergence (mutations) affected the rate of infection with EIDs to the lowest degree.
6. Sarah J. Dolson et al. proposed a numerical technique for measuring the environmental stress of biogeocoenoses, which were situated at different heights above the sea, caused by climate change [127]. Specifically, with their modeling, they identified the effect of phylogenetic clusterization in the biosphere brought on by climate change.

These examples demonstrate the significant heuristic value and predictive force of the SP as a tool for novel EID risk estimation.

## 6. Ethics: The Laws of Biotics

Thus far, we have considered the SP as a natural philosophical concept, a methodological repository and a tool for solving practical tasks. Several words must be said about the SP as an ethical framework.

In an endeavor to re-consider the sustainable development of humanity in evolutionary terms, the authors of the SP introduced the four laws of biotics (thanks to Dr. Norman Johnson, we realized that this was definitely an homage to Isaac Asimov’s laws of robotics):

(1) Humans may not harm the biosphere, by action or inaction.
(2) Humans may not injure any portion of the biosphere, by action or inaction.
(3) Humans may exploit the biosphere up to the limits that do not violate the first two laws.
(4) Humans cannot destroy the biosphere to save themselves.

These simple laws place possible limits on humanity’s perpetual inclination to “subdue the Earth”. Carbonization is one of the most instructive examples of this subdual. The laws allow humans to utilize the “evolutionary commons” [44], a property of ecosystems to accumulate evolutionary potential that can be unleashed in the future. Additionally, the laws may define new vectors of *homo sapiens*’ survival in the situation where more and more EIDs will threaten it.

The current SARS-CoV-2 pandemic caused by breaching all four laws may prove the correctness of the SP’s authors better than anything. Even more importantly, these laws may indirectly indicate the future routes of evolution of *homo sapiens* itself. Indeed, *homo sapiens* becomes a part of the new evolutionary environment, “artificial nature”, and inevitably undergoes adaptation and variation determined by the new conditions. An individual is now forced to adapt evolutionarily to an environment that is no longer nature. The laws of evolution in such an environment are and will be mainly determined by humanity. This “feedback system” resembles what we can usually observe in farming conditions [12,33,34,35]. The following chapter expounds the question.

## 7. The Changed Evolutionary Role of Humans in a Perturbed and Carbonized Biosphere

Perhaps the SP’s most global achievement is demonstrating that, regarding uncontrolled carbon emissions and climate change, we, humans, are dooming ourselves to a new evolutionary role for which we are completely unprepared. To understand this change in the evolutionary role of *homo sapiens*, we invite the readers to take a look at planet Earth and its life forms from a sufficient spatial and temporal distance.

Over periods of several ice ages, species extinctions and interglacial periods, three fundamentally different megabiotopes have developed. They have been expanding and contracting but have retained their highly different specific ecological characteristics [33]:

(a) Boreal coniferous forest ecosystems in the north: today, they form the largest contiguous forest area on the Earth;
(b) Rainforest areas in the Amazon basin, Congo and South/Southeast Asia: The “lungs” of the Earth;
(c) Cold and dry deserts around the poles, hot deserts of the Sahara and some other wasteland areas in Africa, South America and Asia (e.g., Taklamakan Desert, Gobi Desert, Thar Desert, etc.).

Before the spread of modern technical civilization, these natural megabiotopes were essentially only connected with each other by migratory birds (the most populous “carriers” of different parasites: viruses, fungi, protozoa and bacteria), with the transmission and exchange of these viruses, fungi, protozoa and bacteria taking place during bird migrations. The “aggressiveness” of parasites was limited by the necessity for those stowaways to reach their destination on their “means of transport” without destroying this transport [33].

With enormous carbon dioxide emissions in the atmosphere and climate change, many species of migratory birds have become extinct [34,35]. The migration vectors of many other avian species have been distorted by the factors of anthropogenic influence, the main ones being—once again—carbonization of the biosphere and the climate change caused by it [36,37,38]. On the other hand, by the twenty-first century, humans have become the most populous “carriers” for parasite interchange between the three megabiotopes described, not birds, due to the uncontrolled human population growth rate and global lifestyle (limitless tourism, migration and “network society” without boundaries). Therefore, in an evolutionary way, humans now play a role that migratory birds once played in spreading parasite pathogens between the three megabiotopes, the role of “super-spreaders”. This makes humanity a very promising new global “carrier”—and hence a target—for the parasitic causative agents of EIDs. As a result, zoonosis–human diseases switches happen much more often than in the pre-industrial periods of human history [39,40].

However, the immune system of modern humans does not suit this role in a great majority of cases. Due to the impaired environmental conditions and heavy reliance on technology, a modern human is much weaker from evolutionary and immunological points of view than its predecessor of a pre-industrial age.

Today, at least two thirds of the eight billion people on Earth live in almost constant air temperatures of around 20 °C, protected against wind and weather, with sixteen artificially produced hours of daily brightness and food supply, both of which are provided independently of seasonal fluctuations. With people spending most of their time in close physical contact in this largely isolated “feeling-good atmosphere,” they provide an ideal novel host for mutated viruses, fungi, protozoa and bacteria that have been introduced or have grown in megacities themselves [33]. The immune system of this new urbanized species, *homo billionis* (a term coined by Wolfgang Sassin [9,34,35]), which is not sufficiently trained due to a lack of evolutionary challenges, is too weak to prevent the sooner-or-later collapse of this “monoculture of skyscrapers”, i.e., a public health catastrophe in highly urbanized areas, primarily megapolises. This catastrophe would result from the EID burden we are still not ready to carry, as the COVID-19 pandemic has evidently demonstrated.

Acquiring the evolutionary role of EID super-spreaders once played by migratory birds endangers us as a species. After all, today, we know that nearly half the birds never reached their destination due to avian infection, despite their immunity being well prepared for parasitic pathogens [24,33]. In our case, the mortality on the scale of the globe may be much higher than 50%, should a next catastrophic EID that we may overlook have the mortality of, say, Ebola fever and the contagiousness of measles or chicken pox [33,110,111,112].

There are two recipes against such a gloomy fate.

First, we ought to restructure our public care systems so that they should be epidemiologically effective, rather than cost-effective. Second—and most importantly—we have to manage climate change by adopting strict protocols for curbing carbon emissions. According to the SP, even achieving the +1.0–+1.2 °C level regarding the pre-industrial times will not result in stabilizing the biosphere immediately (within the limits of one generation). Evolution works slowly. Darwin once said, “Evolution is lazy” [48]. Humanity launched the generalization of parasitic species by expanding their host range. What we wish to achieve now is their specialization (the opposite process to generalization) and contraction of their host range, with human beings mainly excluded from the list of their hosts. However, the generalization of parasites may proceed at rates a hundred or even a thousand times higher than those of specialization [49]. Achieving the +1.0–+1.2 °C target may result in forming new stable biotic associates with animal mammals as the hosts (without *homo sapiens*), in 1.5–2.5 hundred years [8,12,34]. During this period, *homo sapiens* will inevitably suffer from host oscillations of EID causative agents [39,40]. Regarding this, we may add the threat of novel EIDs to the plant kingdom that would endanger our crops and other farming vegetables due to the host switching of plant parasites from wild flora to agricultural species.

However, targeting +1.2 °C is better than doing nothing. Upon hitting the +2.0 °C limit, the SP predicts that two or three new pandemics (not just local outbreaks) may happen a year. Therefore, efforts to curb CO_2_ emissions are crucial for isolating the EID threat to humanity. Perhaps only our great-great grandchildren will see the result of the efforts to decrease this EID burden, if they are ever achieved. The harm we have already done to the biosphere and to ourselves, as its inseparable part, through uncontrolled carbon emissions and the consequent destruction of our own habitat, is tremendous.

Doing nothing with the decarbonization of the planet will literally cost us our lives. In the end, humans are not the first species on the Earth that lulled itself into a sweet dream of putative evolutionary safety due to its highest place in all food chains. Dinosaurs once also did this.

## 8. Conclusions

The Stockholm paradigm is barely emerging—it is less than ten years old. Very few independent scientific texts have been written thus far on its basics, philosophy, methodology and practicability. Save several book reviews, almost all published works concerned with the SP have been composed by the SP’s creators. To the best of our knowledge, this review is the only large analysis of the SP performed by independent researchers since 2013, when the term was introduced by Daniel R. Brooks. More specifically, we have demonstrated the SP’s significant heuristic value for appraising humans’ evolutionary fate in the distorted and carbonized biosphere that has become the Pandora’s box of emerging infectious diseases. We have also shown how the SP can be a convenient analytical and modeling instrument for evaluating the perspectives of decarbonization.

The SP explains the ways in which climate change causes the appearance of novel EIDs that represent a great threat to the health of individuals and public health systems. The core idea of the SP is that the colonizations of humans are a result of yesterday’s zoonotic pathogens taking advantage of new opportunities with the use of pre-existing capacities; they do not represent the evolution of the new or special capacities of these zoonotic pathogens [128,129]. Therefore, the appearance of EIDs caused by climate change is so fast, and their occurrence is so universal. The SP insists that reducing carbon emissions will be effective in decreasing the EID burden on public health, if it is accompanied by (1) controlling the population growth rate, (2) avoiding mega-urbanization and (3) preventing humans’ destructive interference in the biosphere.

The SP can also be used as a practical tool (modeling, analysis and computation) for assessing the EID risk to public health and connecting the level and distribution of carbon emissions with different characteristics of EID pathogens and diseases themselves. Currently, the SP has not yet been applied to model the epidemiological efficiency and cost efficiency of public health systems in coping with the EID threat. However, it has large potential for it. The future research on SP-based mathematical and numerical algorithms suitable for healthcare systems’ immediate tasks of preventing, controlling and containing EIDs may provide a flexible vehicle for public health.

Finally, we think it useful and advisable to include the SP-based research in the scientific repository of the Intergovernmental Panel on Climate Change.

## Data Availability

Not applicable.
